# Integration of Social, Cultural, and Biomedical Strategies into an Existing Couple-Based Behavioral HIV/STI Prevention Intervention: Voices of Latino Male Couples

**DOI:** 10.1371/journal.pone.0152361

**Published:** 2016-03-30

**Authors:** Omar Martinez, Elwin Wu, Ethan C. Levine, Miguel Muñoz-Laboy, M. Isabel Fernandez, Sarah Bauerle Bass, Eva M. Moya, Timothy Frasca, Silvia Chavez-Baray, Larry D. Icard, Hugo Ovejero, Alex Carballo-Diéguez, Scott D. Rhodes

**Affiliations:** 1 School of Social Work, College of Public Health, Temple University, Philadelphia, Pennsylvania, United States of America; 2 School of Social Work, Columbia University, New York, New York, United States of America; 3 College of Liberal Arts, Temple University, Philadelphia, Pennsylvania, United States of America; 4 College of Osteopathic Medicine, Nova Southeastern University, Fort Lauderdale, Florida, United States of America; 5 School of Public Health, Temple University, Philadelphia, Pennsylvania, United States of America; 6 School of Social Work, University of Texas at El Paso, El Paso, Texas, United States of America; 7 HIV Center for Clinical and Behavioral Studies, Columbia University, New York, New York, United States of America; 8 Lutheran Family Health Centers, New York, New York, United States of America; 9 Wake Forest University Medical Center, Winston-Salem, North Carolina, United States of America; University of Pittsburgh, UNITED STATES

## Abstract

**Introduction:**

Successful HIV prevention and treatment requires evidence-based approaches that combine biomedical strategies with behavioral interventions that are socially and culturally appropriate for the population or community being prioritized. Although there has been a push for a combination approach, how best to integrate different strategies into existing behavioral HIV prevention interventions remains unclear. The need to develop effective combination approaches is of particular importance for men who have sex with men (MSM), who face a disproportionately high risk of HIV acquisition.

**Materials and Methods:**

We collaborated with Latino male couples and providers to adapt *Connect ‘n Unite*, an evidence-based intervention for Black male couples, for Latino male couples. We conducted a series of three focus groups, each with two cohorts of couples, and one focus group with providers. A purposive stratified sample of 20 couples (N = 40, divided into two cohorts) and 10 providers provided insights into how to adapt and integrate social, cultural, and biomedical approaches in a couples-based HIV/AIDS behavioral intervention.

**Results:**

The majority (N = 37) of the couple participants had no prior knowledge of the following new biomedical strategies: non-occupational post-exposure prophylaxis (nPEP); pre-exposure prophylaxis (PrEP); and HIV self-testing kits. After they were introduced to these biomedical interventions, all participants expressed a need for information and empowerment through knowledge and awareness of these interventions. In particular, participants suggested that we provide PrEP and HIV self-testing kits by the middle or end of the intervention. Providers suggested a need to address behavioral, social and structural issues, such as language barriers; and the promotion of client-centered approaches to increase access to, adaptation of, and adherence to biomedical strategies. Corroborating what couple participants suggested, providers agreed that biomedical strategies should be offered after providing information about these tools. Regarding culturally sensitive and responsive approaches, participants identified stigma and discrimination associated with HIV and sexual identity as barriers to care, language barriers and documentation status as further barriers to care, the couple-based approach as ideal to health promotion, and the need to include family topics in the intervention.

**Discussion:**

We successfully adapted an evidence-based behavioral HIV prevention intervention for Latino male couples. The adapted intervention, called *Conectando Latinos en Pareja*, integrates social, cultural, behavioral and biomedical strategies to address the HIV epidemic among Latino MSM. The study highlights the promise regarding the feasibility of implementing a combination approach to HIV prevention in this population.

## Introduction

### Unequal Burden: Latinos and HIV/STIs

In 2013, Latinos in the United States represented 23% of new HIV infections, acquiring more than 10,000 diagnoses, even though they constitute only 16% of the total US population. Latino men who have sex with men (MSM) face an especially heavy burden, representing 81% of all new HIV infections among Latinos [1]. For Latino men infected with HIV, the most common modes of transmission are male-to-male sexual contact and injection drug use [2]. Rates of reported STIs are also higher among Latinos than among non-Latino whites [3]. In particular, predominantly Spanish-speaking Latino MSM are disproportionately at risk for HIV [4–6]. They are less likely to report using a condom at most recent intercourse, seeking or accessing health services, and having a personal doctor and health insurance compared to English-speaking Latino and non-Latino men [7,8]. In addition, evidence suggests that less acculturated Latinos are more likely to engage in risky behaviors because of pre- and post-migration experiences including discrimination, stigma, and isolation [9,10]. Altogether, these data underscore the public health importance of increased efforts to redress the unequal burden of the HIV epidemic shouldered by Latinos.

### Substance Use and Vulnerability to Sexual Risk among Latino MSM

Epidemiological data highlights substance use as a contributing factor to HIV risk among Latino MSM. In a recent study conducted in Indianapolis, IN, we found a high prevalence of alcohol and substance use among recently-arrived behaviorally bisexual Latino immigrant men [11,12]. Similarly, a multisite study among Latino MSM found that heavy alcohol use in the previous six months ranged from 15% in San Francisco to 37% in Chicago, and further documented high use of marijuana use (33% and 27%), poppers (17% and 18%), cocaine (9% and 19%), and speed (19% and 9%) [13]. Research with an internet-based sample of Latino MSM found that 49% of respondents had used club drugs (e.g., cocaine, crystal methamphetamine) in the past 6 months, with poppers being the most popular (32%) [14]. In our recent study with Latino MSM in New York City, most of whom were predominantly Spanish-speaking, 46% of participants screened for high risk alcohol consumption [15]. Alcohol and other substance use and dependence have been closely associated with sexual risk and HIV infection since the beginning of the epidemic [16,17].

In addition to substance use, discrimination, stigma, social isolation, migration experiences, and cultural factors contribute to an elevated risk for many Latino MSM [9,10]. For instance, traditional Hispanic culture stigmatizes and rejects homosexuality, which leads many to shy away from discussing their sexuality, and/or engage in clandestine sexual encounters in which it is more difficult to use HIV protection [18–22]. “Machismo” norms prescribe that men must avoid feminine behaviors (e.g., being penetrated) and prove their manhood by having multiple partners and unprotected sex [23–25]. Latino MSM may feel further compelled to engage in risk behaviors to compensate for negative perceptions about their sexual orientation as well as internalized homophobia [26,27]. For many, the stress of not having legal U.S. residency status, the challenges of navigating different cultural contexts and the loneliness that arises through carrying “secrets” and estrangement from families leads many Latino MSM to have unprotected sex as a source of comfort and relief; such patterns are exacerbated by the fact that many Latino MSM may also turn to alcohol and other substance use to cope with marginalization, further increasing their risk of engaging in unprotected sexual acts [28,29]. These data underscore the need to implement cost-effective sexual risk and substance use harm-reduction interventions and programs for Latino MSM.

### HIV Prevention Interventions for Latino MSM

Some behavioral interventions have been developed specifically to address HIV infections among Latinos [30,31], including some targeting predominantly Spanish-speaking MSM [32–34]. These interventions have examined behavioral and structural barriers to HIV care and testing, including unprotected anal intercourse and immigration status obstacles to accessing health services. Culturally congruent interventions using social networks and support groups among predominantly Spanish-speaking Latinos have been successful in reducing HIV risk behaviors [30,35]. The Hombres Ofreciendo Liderazgo y Ayuda (HOLA; Men Offering Leadership and Help) intervention relies on social networks established by predominantly Spanish-speaking Latino MSM in rural areas to deliver HIV prevention and treatment, and takes into consideration barriers to care, including immigration status and the anti-immigration rhetoric in the Southeastern US. One of HOLA’s key components is to address cultural values and build on community assets, including existing informal social networks, to address health challenges affecting Latinos [32]. However, HOLA and other interventions have not incorporated newly available biomedical strategies, including PrEP and HIV self-testing kits. Thus, there remains a great need to investigate the effectiveness of combination prevention strategies: integrating evidence-based and impactful behavioral, biomedical, and structural intervention strategies to reduce HIV incidence, while providing the tools to address the larger HIV epidemic [36,37].

Biomedical approaches draw on research from medical and public health fields in order to address a range of factors in HIV prevention. In the US, four such approaches are being promoted specifically for MSM: treatment as prevention (TasP) [38]; HIV self-testing kits [39,40]; pre-exposure prophylaxis (PrEP) [3,41,42]; and non-occupational post-exposure prophylaxis (nPEP) [43–45]. Recent developments in the use of PrEP and TasP have shown promising results. Oral PrEP was found efficacious in several studies with men who have sex with men (MSM). The iPrEx study, first published in 2010, demonstrated a reduction in relative risk (RRR) of HIV seroconversion of 44% for continuous PrEP with tenofovir disoproxil fumarate (TDF) and emtricitabine (FTC) in MSM. The efficacy of PrEP has been confirmed for continuous PrEP in the PROUD study and for intermittent PrEP in the IPERGAY study (RRR = 86% in both studies) [46]. The HPTN 052 trial with serodiscordant, predominantly heterosexual couples demonstrated that the provision of early ARV treatment reduced HIV transmission by 96% relative to delayed treatment (i.e. provision of ARV once infected partners’ immune systems were declining). This has encouraged the development of TasP programs [47]. However, all biomedical strategies depend on 1) patients’ acceptance and adherence, and 2) healthcare providers’ belief that these interventions are needed. Neither is guaranteed. A recent study found that providers disagreed as to which patients were most appropriate to receive PrEP, and that many providers believed that current models of care assume routine office visits and are thus not well suited for prescribing PrEP to MSM and transgender women [48]. There is growing interest in expanding public health approaches that address social and structural factors to tackle HIV prevention [49]. Combining biomedical approaches with social and structural components ensures an even more comprehensive and targeted intervention [50]. However, successful integration of biomedical strategies in behavioral interventions will depend on not only promoting participants’ knowledge of these new strategies and their potential worth as HIV prevention methods, but also providers’ understanding that these are viable and important new strategies for reducing HIV in vulnerable populations.

Some initiatives are promoting these new biomedical prevention tools to tackle the HIV epidemic. For example, New York Governor Andrew Cuomo launched the AIDS Free New York initiative, which aims to eliminate AIDS in the state by 2020 [51]. The initiative is grounded in: 1) identifying undiagnosed persons with HIV and linking them to health care; 2) linking persons diagnosed with HIV to health care and utilizing anti-HIV therapy to maximize viral suppression; and 3) providing access to PrEP for high-risk, HIV-negative persons.

Building on existing behavioral interventions and recognizing the need for an approach that integrates prevention and treatment [52–57], we wanted to assess how biomedical strategies could be integrated into a couples-based behavioral HIV prevention intervention for predominantly Spanish-speaking Latino MSM and their same-sex partners—a population at high risk of HIV infection. In our search for intervention models, we identified *Connect ‘n Unite* (CNU) as particularly promising. CNU is a couple -based intervention for substance-using Black MSM. Its four sessions address a range of topics including communication and support within relationships, strategies for self-care, and education about HIV/AIDS and risk behaviors. This intervention has been shown effective in promoting condom use and decreasing harmful substance use in Black male couples [58–60]. However, CNU did not incorporate newly available biomedical tools such as PrEP and nPEP.

For this study, we identified, assessed, and explored how new biomedical approaches could be incorporated into an HIV/STI behavioral intervention for Latino male couples. Building on the content of CNU, we sought to incorporate biomedical approaches into existing sessions. Moreover, recognizing that minority MSM couples are not a monolithic population, we sought to adapt CNU curricula specifically for Latinos to ensure that relevant cultural (e.g. machismo norms) and structural factors (e.g. immigration concerns) were addressed within intervention sessions. We conducted focus groups with Latino MSM couples as well as providers who serve this community, and applied their insights to develop a culturally specific, behavioral and biomedical prevention intervention called *Conectando Latinos en Pareja* (CLP). Details of the adaptation process have been published previously (58). For the purposes of this paper, we focus specifically on the incorporation of biomedical prevention strategies into CLP sessions. To our knowledge, ours is the first study that assesses the potential of a combined biomedical, social, and structural approach to reducing HIV transmission in this population.

## Methods

The adaptation process included five steps: 1) engaging community stakeholders; 2) capturing the lived experiences of Latino male couples; 3) identifying intervention priorities; 4) integrating the original intervention’s social cognitive theory into a relationship-oriented, ecological framework for Latino male couples; and 5) adapting intervention activities and materials. Findings related to the adaptation process have been published elsewhere [61]. For this particular manuscript, results emphasize the focus group data that helped us identify, assess, and explore how new biomedical approaches could be incorporated into an adapted HIV/STI behavioral intervention for Latino gay couples.

### Ethics Information

The New York State Psychiatric Institute review board approved the research. In addition, we obtained a National Institutes of Health Certificate of Confidentiality in accordance with the provisions of section 301(d) of the Public Health Service Act 42 U.S.C. 241(d) to further protect the privacy of research subjects. All potential participants provided verbal informed consent to take part in the brief screening interview to determine eligibility and willingness to participate. We requested waiving documentation of written informed consent for screening only, which may be obtained if “the research presents no more harm than minimal risk of harm to subjects and involves no procedures for which written consent is normally required outside of the research context” [45 CFR 46.117(c)]. All the couples provided written consent to take part in the focus groups.

### Recruitment

#### Latino Male Couples

Couples were recruited through direct contact, social media networks such as Facebook and Grindr, and via community-based organizations serving the needs of predominantly Spanish-speaking Latino MSM in the five boroughs of New York City. Social media served as an especially powerful tool for recruiting participants. We relied on community stakeholders to develop materials and shape the wording and messages of social media postings. Stakeholders were introduced to methodological approaches in order to guide the development of social media recruitment materials. Further details have been published elsewhere [62].

Purposive stratified sampling [63] was used to diversify the sample in terms of age, sexual and gender identity., country of origin, race/ethnicity, HIV status, duration of the relationship, relationship dynamics, immigration/documentation status, prior links to health agencies, and previous involvement in HIV research. While all participants responded to a call for research with Latino MSM in same-sex partnerships, some Latina transgender women in relationships with men expressed interest in participating. Rather than exclude them, we accepted their self-assessment as members of this community. Potential participants were invited to complete a phone or in-person questionnaire for a larger, mixed methods study on Latino MSM. Questions addressed a range of issues including but not limited to alcohol use, mental health, sexual behaviors, relationship status, and demographic characteristics. The screening instrument enabled us to recruit a diverse sample of key informants for our intervention adaptation sessions. Focus group participants were informed that they would be assisting with an adaptation effort, helping to develop an intervention rather than receiving the intervention themselves.

Altogether, we recruited 20 couples for this study (i.e. 40 individual participants). We divided these participants into two separate cohorts, composed of nine and 11 couples, each of which took part in three focus group sessions. This made the focus groups more manageable in terms of facilitation, note-taking, and data analysis. Additionally, running the adaptation twice provided greater breadth of findings; each cohort provided some insights, some perspectives that the other did not. While we worked to ensure that both cohorts were diverse in terms of demographics and relevant background, and roughly equivalent in terms of sexual and substance use risk behaviors, we were also mindful of potential risks of discomfort. For example, after recruiting two transgender women and their male partners, we elected to place both of these couples in Cohort 1. Rather than ensure transgender representation in both cohorts, we wanted to reduce the chances of our transgender participants feeling isolated or “othered” in focus group sessions.

#### Eligibility Criteria for Couples

Couples were eligible for the *Conectando Latinos en Pareja* study if they met the following criteria: 1) both partners were 18 years or older; 2) both considered the other as their “main partner,” which was operationalized as (a) a man or transgender woman (male-to-female) with whom the participant has had an ongoing sexual relationship over the prior 3 months, (b) considered a “boyfriend, girlfriend, domestic partner, spouse, ongoing lover, or regular partner,” and (c) stated intention to remain together for at least 12 months; 3) at least one partner self-identified as Latino/a or Hispanic (i.e., a native or inhabitant of Latin America; a person of Latin American origin living in the United States); 4) at least one partner had limited English proficiency, and both partners were proficient in Spanish; 5) at least one partner reported one or more unprotected acts of anal intercourse in the past year, within or outside of the relationship; and 6) at least one partner reported using illicit substances or other drugs/substances not prescribed by a doctor that change mood or thinking in the past 3 months, or screening for high-risk alcohol consumption. High-risk alcohol consumption was operationalized as: binge drinking (5 or more alcoholic drinks on the same occasion on at least 1 day in the past 30 days) or heavy drinking (5 or more alcoholic drinks on the same occasion on each of 5 or more days in the past 30 days). Criterion 4, related to limited English proficiency, was assessed by asking the following: Considering English and Spanish, mark the option that best represents your ability to read, speak, and write in these languages. Likert responses include: 1 = Only Spanish; 2 = Spanish better than English; 3 = Both equally; 4 = English better than Spanish; 5 = Only English. Participants answering 1 or 2 met the limited English proficiency criteria.

Couples-based interventions raise concerns about participant safety, specifically for victims of intimate partner violence (IPV). Experiences of IPV were assessed using the Revised Conflict Tactics Scale [64–67]. Those who disclosed IPV were asked the following question to determine eligibility: Would participating in a study that talks about drug and alcohol use, sex, or relationship issues cause you to be concerned about [partner’s first name] putting you in danger?. If a participant answered “yes”, he or she was prompted with a follow-up question: Given this concern, what could we do to maximize and monitor your safety if you agreed to participate? Finally, we asked: If we took these precautions (i.e. strategies identified by the participant and principal investigator [PI] to mitigate safety concerns), would you feel safe enough to participate in the study where we talk about drug and alcohol use, sex, and relationship issues with [partner’s first name]? Our rationale to include couples who reported IPV, but who were not in fear or danger, was based on feedback from community stakeholders indicating that IPV affected some of their clients. This decision also served to diversify the sample of couples based on their lived experiences. Potential participants (in conjunction with their main partners) would have been excluded as a couple if: 1) either partner reported the occurrence of one or more incidents of IPV within the relationship in the past year and expressed safety concerns about participating, or 2) either partner had a language or cognitive impairment that would prevent comprehension of study procedures as assessed during the consent process. Using these criteria, we did not need to exclude any participants based on reports of IPV.

#### Health and Social Services Providers

The principal investigator (PI) and co-principal investigators solicited health and social service providers or volunteers from prominent organizations working with Latino MSM to participate in the providers’ session, specifically those who provided direct services and/or referrals to Latino male couples. The PI contacted directors from local organizations via phone, email or face-to-face contact to identify staff participants. Ten providers and/or volunteers from a wide range of organizations, including the Latino Commission on AIDS, Aid for AIDS International, and Hispanic AIDS Forum, participated in the intervention adaptation session.

### Screening Measures

Demographic characteristics included age; country of origin; sexual identity; language spoken, written, and read; and recruitment method.

High-risk alcohol consumption was assessed using the National Institute on Alcohol Abuse and Alcoholism assessment: binge drinking (more than four alcoholic drinks on the same occasion on at least 1 day in the past 30 days) and heavy drinking (more than four alcoholic drinks on the same occasion on 5 or more days in the past 30 days).

Substance use was assessed by sequentially asking participants to report whether during the prior 3 months they used methamphetamine, marijuana, cocaine in various forms, heroin/other opiates, tranquilizers, other club drugs and stimulants, and nonprescribed erectile dysfunction drugs.

Sexual risk behavior was assessed by asking participants to report the number of male sexual partners in the previous three months. This was followed by a series of questions that prompted the respondent to indicate the number of anal intercourse episodes and the times condoms were used during this time period.

Relationship characteristics were assessed by inquiring about length of relationship (dichotomized as 1–12 months or more than 12 months) and about any experience of IPV.

Health services providers completed a brief screening instrument before the session with questions about gender; professional discipline; education; language spoken, written and read; percentage of Spanish-speaking clients; whether or not they had received any training in serving the health needs of Latino gay couples; major challenges affecting Latino gay couples; barriers with implementing the adapted intervention; and perceptions about the inclusion and integration of biomedical approaches in an adapted, couple-based, HIV/STI prevention intervention.

### Focus Group Procedures

We held three focus groups each with two cohorts of Latino male couples. Each session lasted between two and three hours, and was audio recorded and conducted in Spanish. Participants provided informed consent and were given the option to use pseudonyms to protect their privacy. The first author and a co-author led and facilitated the discussions following a pre-established list of topics while five note-takers captured nonverbal information and ensured that all comments were accurately attributed. All discussions with Latino couples were conducted in Spanish. We continued to elicit dialogue from the focus groups until no new information resulted. Facilitators and note-takers debriefed after each focus group, and their reflections were recorded. These records were later supplemented by the principal investigator (PI), who provided extensive additional notes after listening to audio recordings of each session.

The first two focus group sessions involved “theater testing” of core components of the intervention including key activities, such as overviews of available biomedical prevention strategies, and homework assignments. The open-ended questions for couples explored participants’ knowledge and perceptions about biomedical strategies, including nPEP, PrEP, and HIV self-testing kits; their experiences in accessing these tools; and their suggestions of how to incorporate them into a couple-based behavioral HIV/AIDS prevention intervention. Sample questions are grouped by domains in Table 1. We tailored focus group questions to the particular needs of couples based on partners’ HIV statuses. For example, we asked whether individuals who reported being HIV negative would be willing to use a self-testing kit to reassure a partner, and we asked serodiscordant couples whether they thought PrEP might be an appropriate resource for them. In the third and final session, we solicited feedback geared towards increasing the feasibility of future, larger scale studies. More specifically, we sought insights regarding recruitment and retention, logistical barriers to participating in the intervention, and strategies for addressing safety concerns and any adverse events that might arise. For both cohorts, Sessions 1 and 2 occurred on the same day; Session 3 occurred on the following day. Retention was 100% among those recruited. Individuals received $40 for attending each of three focus groups, for a total of $120 for their participation.

**Table 1:** Sample Questions Regarding Biomedical and Structural Approaches.

Domains	Sample Questions
Information and Knowledge Regarding Biomedical Interventions	*Have you heard about Pre-Exposure Prophylaxis (PrEP)?*
	*Do you think it is effective? If so*, *in what ways?*
	*What do you know about non-occupational Post Exposure Prophylaxis (nPEP)?*
	*What do you know about the HIV self-testing kit?*
Motivation and Feasibility of Incorporating Biomedical Approaches into the Existing Behavioral Intervention	*How do you decide which HIV test and/or biomedical approach is best for you?*
	*What reasons would you have for using the HIV self-testing kit and/or the biomedical approaches?*
	*What reasons would you have for NOT using the HIV self-testing kit and/or biomedical approaches?*
	*Would you be willing to test yourself to reassure your partner that you are HIV-negative using the HIV self-testing kit? Explain why*.
	*How feasible would it be to use an HIV self-testing kit like this or biomedical approaches with your partner or a variety of partners?*
	*How would you discuss using the test or biomedical approaches with your main partner or a variety of partners? Do you think that PrEP would be useful for serodiscordant couples?*
	*How can we incorporate these biomedical tools into the existing couple-based HIV behavioral intervention?*
Perceived Challenges and Structural Barriers To Accessing Care and Services	*Tell me about any recent experiences in which you think your immigration status has limited or prevented you from accessing and utilizing health services or other resources in the community?*
Feasibility of Incorporating Strategies to Cope with Perceived Challenges and Structural Barriers to Participating in the Intervention	*How might this intervention address these concerns?*
	*Should we incorporate focus groups and media components as part of the behavioral intervention?* *If so*, *how can we incorporate these structural aspects into the couple-based HIV behavioral intervention?*

We conducted one focus group with providers. Providers were asked about what type of biomedical strategies they saw as more useful for a couple-based behavioral HIV prevention for Latino gay couples. We used open-ended follow-up questions such as, *Tell me more about how would you like to see PrEP in a couple-based HIV prevention program?*—to increase the likelihood of gaining a complete and accurate understanding of the combination prevention approach. We continued to elicit dialogue from the focus group until no new information resulted.

Couples’ and providers’ focus groups were conducted at the School of Social Work at Columbia University.

### Data Analysis

We used a qualitative, descriptive research design [68,69]; focus group data were analyzed, and themes were developed using content analysis [70,71]. Content analysis includes creating smaller segments of the data and then placing a code within each segment. Audio recordings were transcribed verbatim, and codes were identified inductively by topic or question. Significant statements were considered in the context of the group conversation to assess missing information and interpret discrepancies. Codes were grouped by emergent themes through a reflexive and interactive process, and transcripts were re-read to evaluate the accuracy of the identified themes. Data collected from both cohorts of Latino MSM were pooled into a “couples” word document, as we were more interested in assessing the range of participants’ perspectives than identifying discrepancies and commonalities between cohorts. The PI, co-investigators, note-takers, and consultants held three meetings to analyze data and reach consensus about coding strategies, emergent themes, and disagreements regarding the interpretation and classification of various passages.

## Results

### Characteristics of Latino Male Couples and Health Providers

Our study sample consisted of twenty couples (n = 40) in two cohorts, Cohort 1 (9 couples, n = 18) and Cohort 2 (11 couples, n = 22). Participant characteristics can be found in Table 2. The mean ages were 35.61 and 39.23 respectively. A large number of study participants were from Mexico, (Cohort 1 n = 6, 34% and Cohort 2 n = 5, 23%), self-identified as gay (Cohort 1 n = 15, 83% and Cohort 2 n = 18, 82%), and were predominantly Spanish-speaking (Cohort 1 n = 16, 89% and Cohort 2 n = 19, 86%). We further noted high rates of sexual risk and substance use risk behavior among our 40 Latino MSM and Latina transgender women participants. The majority screened for risky alcohol consumption, and approximately 40% reported using party and club drugs within the previous three months. More than 60% reported engaging in unprotected acts of anal intercourse with the previous three months. Characteristics of health service providers (n = 10) can be found in Table 3. Quotes include pseudonyms to protect the identity of participants.

**Table 2:** Descriptive characteristics of Latino male couples.

	M ± SD or n (%)	
	Cohort 1	Cohort 2
	(N = 9 couples; 18)	(N = 11 couples; 22)
Age (mean)	35.61 (8.16)	39.23 (8.78)
18–24	1 (6)	1 (5)
25–34	8 (44)	5 (23)
35–44	5 (28)	8 (35)
45 or older	4 (22)	8 (37)
Country of Origin		
Mexico	6 (34)	5 (23)
Central American		
Salvador	0	1 (4)
Honduras	3 (17)	1 (4)
Guatemala	1 (5)	1 (4)
South American		
Ecuador	0	1 (4)
Colombia	0	6 (27)
Peru	0	1 (4)
Venezuela	3 (17)	1 (4)
Caribbean		
Cuba	3 (17)	2 (11)
Puerto Rico	1 (5)	2 (11)
Dominican Republic	1 (5)	1 (4)
Sexual and Gender Identity		
Gay	15 (83)	18 (82)
Bisexual	1 (6)	4 (18)
Transgender/Transexual	2 (11)	0
Primary Language		
Only Spanish	7 (39)	8 (36)
Spanish better than English	9 (50)	11 (50)
Both equally	2 (11)	3 (14)
Recruitment Venue		
Community-based organization	6 (33)	4 (20)
Social Media (Facebook, Grindr)	8 (45)	2 (10)
Friend/Couple Referral	4(22)	16 (70)
High-risk Alcohol Consumption		
None	6 (33)	5 (23)
Binge	8 (45)	9 (41)
Heavy	4 (22)	8 (36)
Substance Use in Past 3 Months		
Marijuana	3 (17)	7 (32)
Powder cocaine	0	3 (15)
Heroin	1 (6)	0
Party and club drugs	7 (39)	10 (45)
Sexual Behaviors in Past 3 Months		
Anal sex acts (mean)	34.83 (27.83)	23.73 (20.15)
Unprotected anal sex acts (any)	14 (78)	14 (64)
Multiple sexual partners	11 (61)	10 (45)
Length of relationship		
1–12 mo	8 (45)	9 (41)
More than 12 mo	10 (55)	13 (59)
Reported Intimate Partner Violence	4 (22)	4 (18)

**Table 3:** Characteristics and Perspectives of Health Service Providers.

Characteristics	M ± SD or n (%)
Age (mean)	35.80 (8.66)
Gender	
Male	8 (80)
Female	2 (20)
Professional discipline	
Research	3 (30)
Public health professional	3 (30)
Wellness and HIV Tester/Counselor	1 (10)
Case Manager	2 (20)
Outreach Coordinator	1 (10)
Education	
Some college	2 (20)
Bachelor's Degree	2 (20)
Advanced Degree	6 (60)
Primary Language	
Spanish better than English	3 (30)
Both equally	4 (40)
English better than Spanish	3 (30)
Spanish-speaking clients	
0–25%	1 (10)
25–50%	1 (10)
50–75%	0
75–100%	8 (80)
Training on serving Latino gay couples	2 (20)
Challenges identified for Latino MSM	
Legal issues (e.g., documentation status, marriage equality, police harassment)	9 (21)
HIV and STIs	6 (15)
Mental health	5 (12)
Intimate partner violence	5 (12)
Childhood sexual abuse	7 (17)
Substance use and/or excessive alcohol consumption	7 (17)
LGBT Stigma	1 (3)
Discrimination within LGBT community	1 (3)
Barriers to implementing adapted intervention	
Limited provider time with patients	5 (31)
Uncertainty about intervention requirements	6 (37)
Limited training	3 (18)
Recruitment strategies	1 (7)
Legal issues and concerns	1 (7)
Support for biomedical interventions	
Pre-exposure prophylaxis (PrEP)	10 (26)
Post-exposure prophylaxis (PEP)	6 (16)
Medical male circumcision	2 (6)
HIV self-testing kit	5 (13)
Male and female condoms	5 (13)
Treatment as prevention (TasP)	9 (23)

### Knowledge of and Reception to Biomedical Strategies among Latino Male Couples

After reviewing various biomedical strategies in focus group sessions with couples, facilitators led extensive discussions about the potential benefits of incorporating such tools into interventions, strategies for raising awareness and promoting appropriate use, and potential barriers to community uptake. Only one of the 40 participants indicated prior knowledge of nPEP and PrEP, having learned of them through his involvement in outreach initiatives within a community-based organization in New York City. Participants mentioned the need to introduce information about nPEP and PrEP earlier in the sessions of the behavioral intervention and provide the option of using these biomedical strategies after a few sessions. One of the participants stated that *Medications need to be accompanied by information and instructions on their proper use* (Juan).

The majority of participants stressed the importance of information and awareness and saw in themselves the potential to become agents of social change in their communities through the use of biomedical strategies. One participant expressed that, *Information is power*, *power creates change*. *We need to speak of the importance of taking knowledge and information from these sessions to spread to others*, *including friends*, *family and neighbors* (Mario, ID40). However, one participant highlighted the need to go beyond just spreading information. The participant explained, *We need to generate interest in nPEP and PrEP not just by providing information but by building awareness* (Gabriel). Consistent with this statement, many participants perceived PrEP and nPEP as particularly promising. In particular, participants highlighted the importance of PrEP for serodiscordant couples while developing methods of delivery that mitigate adherence issues. One participant asked, *What about the needs of serodiscordant couples?* *We need more information about PrEP*. *We should be talking about these things*. *Couples are tired of using condoms and information about biomedical tools can help a lot of people* (Carmelo). Such feedback further pointed to the significance of sexual desires and practices among Latino MSM. Couples who are “tired of using condoms” may reject them as a prevention strategy, but may be more amenable to alternatives such as PrEP that do not affect or interfere so directly with sexual encounters.

Regarding HIV self-testing kits, only two participants reported prior knowledge of this resource and its availability on the market. After a formal introduction to the utility of the HIV self-testing kit, all of the participants mentioned that they would like to have access to it by participating in the intervention. One participant mentioned, *If I had access to a self-test 20 years ago when I saw a friend of mine die of AIDS*, *I would have gotten tested a long time ago*. *But it was fear and shame of having a doctor tell you to your face ‘You have AIDS*, *you faggot (puto)’ that kept me from going and getting tested* (Juan). The participant highlighted the importance of the HIV self-testing kits, in particular the potential for kits to alleviate barriers to HIV testing and care, including fears of stigma and discrimination by health providers. However, as in the case of nPEP and PrEP, participants stressed the need for information. *It is good to have the self-test available at any moment*, *and people can take it or not take it whenever they want to–as long as the test is accompanied with information and referrals to counseling services* (Raul). Other participants drew attention to the issue of affordability of HIV self-testing, and the potential benefits of providing kits through the intervention.

Facilitators provided information about TasP to both cohorts, and solicited feedback about this biomedical tool. Counter to expectations, this generated minimal discussion. Participants seemed overall far more interested in PrEP, nPEP, and self-testing kits.

Providers shared couples’ enthusiasm for biomedical prevention strategies. However, providers demonstrated familiarity with the overall lack of awareness of PrEP and other biomedical tools among Latino male couples, and insisted that such resources be provided along with culturally appropriate information. One elaborated, *We need to educate participants from the beginning of the intervention about biomedical interventions*, *in particular through adherence information*. *PrEP should be an incentive towards the latter part of the study*, *once participants have received information*, *knowledge*, *and counseling around PrEP*. *However*, *PrEP shouldn’t be passed out like candy and should especially be promoted for serodiscordant couples* (Provider). To be clear, this individual was not suggesting that providers withhold treatment or demand that MSM go through extensive training or otherwise face additional barriers. The consensus was that PrEP is a particularly promising prevention tool for Latino MSM, but that this potential cannot be realized if individuals lack basic information about proper use, potential benefits, and limitations (e.g. side effects, and the fact that PrEP is an HIV prevention tool and will not protect against STIs).

### Building Culturally Sensitive and Socially Grounded Interventions

Couple participants identified the need to work with agencies and hospitals to promote greater tolerance and education regarding gender issues. Stories and anecdotes documented structural stigma and discrimination in many of the care agencies that serve Latinos. All participants saw a need to incorporate a gender sensitivity component into the intervention as well as establish partnerships with health care providers to promote awareness about gender sensitivity and gender related issues. For instance, one self-identified transgender participant mentioned, *When I changed my name*, *a nurse insisted on calling me ‘he’ and that made me uncomfortable*, *since I consider myself a woman* (Maria). Another participant further explained, *I have felt discriminated before at medical agencies because of my gender identity*. *The doctors are not helpful at all* (Carlos).

Participants also identified couple-based behavioral interventions as key to addressing and overcoming couple-based issues and challenges, including negotiation, communication skills, and creating safe spaces in the community. One participant highlighted the importance of a facilitator in the couples’ context: *Sometimes it’s not easy to talk to each other and express our feelings–sometimes you need a third party*, *a moderator*, *who is impartial* (Aristobulo). Another participant elaborated on the importance of the couple-based approach: *I have been to a lot of workshops but they have always focused on individuals and single gay men*, *and this is one that focuses on the couple context as an approach to promote health*, *and this was interesting to me* (Yusmani).

Other participants identified documentation status as a barrier to accessing services and emphasized the need to incorporate referrals that better address barriers to care due to documentation status. Even those who were documented but were not citizens, reported being afraid to seek help and support due to the misunderstanding that this was going to affect their legal status. One participant noted, *Many people won’t go and seek out services because they are afraid it will affect their immigration status–for fear of being found out–as an undocumented person*, *they don’t go out to find help* (Esteban). Another further elaborated, *I feel that immigration status is always an important issue for couples and people*, *and it can be difficult to be healthy or have access to health care because of it [immigration status]* (Torrealba). Another participant added, *Groups like these can help in spreading the word about spaces and health resources in the community that we can access–regardless of immigration status* (Roman). Others highlighted the potential of the behavioral intervention to promote social change regarding anti-immigrant rhetoric. One participant expressed, *We can fight against fears related to immigration by spreading information and letting people know what services and resources are out there* (Raul).

Most of the participants suggested that the interventions incorporate behavioral tools and strategies to tell their family members about their sexual orientation (i.e. to “come out”). While family can be an important source of support for LGBT Latinos, most participants reported struggling to get support, and many shared anecdotes of losing support from their families. Others expressed issues and hesitation in coming out to family members. A participant shared, *I have a lot of friends who are here alone*, *undocumented and they can’t communicate with family because their family doesn’t accept the fact that they are gay*. *This is another challenge that is specific to our community and should be addressed with the project* (Ignacio).

Similarly, providers identified barriers preventing predominantly Spanish-speaking Latino MSM from accessing health services, including ARV treatment. Major barriers identified include limited English language skills, documentation status, stigma associated with HIV and sexual identity, and homophobia within and outside the Latino community which creates hesitation to seek out HIV testing services. Providers further insisted that efforts to address these barriers must be deliberate and thorough. For instance, one emphasized the need for comprehensive information that is both culturally and linguistically appropriate regarding biomedical interventions, as well as the need for a holistic provider client-relationship: *Just because something is written in Spanish does not mean it is culturally competent*. *Beyond speaking Spanish*, *a provider must be caring to clients and meet their needs and be culturally competent while still dealing with paperwork and the demanding environment of care provision* (Provider).

Providers also identified structural barriers to providing PrEP in hospitals including bureaucracy, mistreatment, and discrimination. Many providers shared stories of discrimination faced by their clients as they sought out services in institutions such as hospitals in Queens, the Bronx and Brooklyn. In order to avoid or reduce such barriers, providers suggested that we offer biomedical interventions in non-traditional care settings such as community based organization and advocacy centers. Providers also suggested that linkage to biomedical interventions could be short-term with intensive care management in order to prevent fatigue and minimize bureaucratic obstacles. This approach would be most appropriate among individuals who undergo periods of high risk. For example, HIV-negative MSM might face an elevated risk of HIV infection when pursuing casual sex, or engaging in sexual relationships with multiple partners, particularly if they engage in condomless anal intercourse. These same individuals might face a relatively low risk in the event that they enter into seroconcordant, monogamous relationships.

### Towards an Integrated and Comprehensive Approach

Latino couples stressed the need for comprehensive interventions. Rather than considering sexual risk in isolation, they emphasized that alcohol and other substance use, relationship dynamics, and sexual behaviors interact with one another: *When you are talking about unwritten rules in the couple*, *you can’t separate domestic violence*, *drugs*, *alcohol*, *and unprotected sex*. *You have to put them all together* (Juanelo). This suggests that simply providing information about and access to PrEP and other tools will be insufficient to address the HIV epidemic among Latino MSM. We must design interventions that address additional factors, including intimate partner violence and alcohol and other substance use. This is consistent with previous literature concerning relationships among substance use behaviors, sexual behaviors, and HIV risk (13–17, 20).

Providers echoed these concerns, and agreed that a combination approach to HIV prevention was the ideal prevention strategy for Latino male couples. Several mentioned the need to address unique characteristics, including substance use and mental illness, when promoting nPEP, PrEP, and HIV self-testing kits. In addition to ensuring that interventions meet a broader range of needs among Latino MSM, providers suggested that interventions designed to address behavioral, social and structural issues would further serve to increase access and adherence to biomedical interventions.

## Discussion

A comprehensive approach that combines biobehavioral strategies to HIV prevention offers the potential to address the epidemic among vulnerable populations [37,72,73]. Although biobehavioral prevention is not a new idea, many questions persist, including best strategies for systematically implementing new biomedical strategies and how potential participants feel about these approaches to HIV prevention. Our study sheds light on community members’ and providers’ perceptions and impressions. Despite the concerns surrounding the use of new biomedical strategies such as nPEP, PrEP, and HIV self-testing kits, including the side effects of nPEP and PrEP medications [41,74], Latino MSM couples and provider participants highlighted the importance of offering tools after providing comprehensive information about them. Similar to other studies exploring barriers among recently-arrived immigrants with limited English proficiency [75,76], providers identified language barriers as among the major obstacles to obtaining care and suggested a client-centered approach with the behavioral intervention to increase access and adherence to PrEP.

While research has documented a range of barriers in health care, including discrimination and a lack of cultural and gender sensitivity, our study provides further evidence that these disparities persist in New York City, particularly in heavily populated immigrant areas such as Queens and the Bronx. This is noteworthy, given that New York City is often perceived as leading the way in biobehavioral HIV prevention efforts. Indeed, the NYC Department of Health and Mental Hygiene has demonstrated a commitment to promoting PrEP for at-risk populations, Yet Latino MSM, and perhaps other marginalized communities within the broader category of “at-risk groups,” have not been reached. Couples and providers identified structural stigma and discrimination, documentation status, and family issues as barriers to care with important impacts on health outcomes. Working with couples was perceived as particularly promising for overcoming such challenges as condom-use negotiation and adherence to nPEP and PrEP. Providers offered further suggestions regarding how the behavioral intervention could alleviate barriers to care, particularly in regards to documentation status and access to PrEP and nPEP.

In order to effectively raise awareness of biomedical prevention strategies, and promote community uptake and treatment adherence among Latino MSM and other marginalized populations, we must seek the insights of community members and providers who serve them. We need to be mindful of the risks of HIV prevention fatigue [77,78], tailor prevention messaging to specific at-risk groups, and reassess strategies for engaging these populations in HIV prevention and treatment. Furthermore, we must not assume that all Latino MSM and Latina transgender women are aware of newly available biomedical strategies. Only one out of 40 participants in our couples focus groups reported prior knowledge of PrEP and nPEP. The *Conectando Latinos en Pareja* intervention addresses this issue through providing overviews of these and other prevention tools. Such overviews should be incorporated into other interventions that target populations at an elevated risk of HIV acquisition, as well as case management and other care strategies. Media prevention campaigns might further raise awareness through offering information about PrEP and nPEP, and encouraging MSM to contact providers about these resources.

An additional finding of this study, perhaps minor but nonetheless noteworthy, was that new biomedical strategies could help to incorporate concerns regarding sexual pleasure in HIV prevention. Sexual pleasure must be taken more seriously and addressed more realistically in public health approaches to HIV prevention, particularly for sexual and gender minorities. Our participants reinforced these concerns, stressing not only the importance of providing culturally appropriate care, but of considering the sexual desires and practices of Latino MSM couples when designing and administering interventions. Couples (and single MSM) who are “tired of using condoms,” but invested in HIV prevention, have much to gain from tools such as PrEP.

Several recommendations emerged from both participants and providers in regards to moving patients through the continuum of HIV care and working to ensure that those who are negative remain so: patient support services should be employed, including assistance with navigating the complex health infrastructure, from actual and perceived barriers to getting tested to those associated with the lack of insurance in accessing ARTs; the need to produce and distribute culturally appropriate healthcare resources, so that care is inclusive of target populations and addresses their particular needs (e.g. considering variation within the broader category of Latino MSM, such as a transgender Latina undocumented immigrant living in Jackson Heights, a heavily populated Latino enclave in NYC, trying to access resources in Chelsea, a neighborhood catering the health needs of more affluent gay men); and verbal encouragement and innovative methods (including social media) from clinic staff in promoting health care utilization and retention.

For any HIV prevention intervention to be effective, it must empower and be used effectively by the target population. Integration of effective biomedical components into behavioral interventions is critical to the successful prevention of new infections, particularly among vulnerable groups and those with limited access to the current care infrastructure. Like other long-standing biomedical cornerstones for HIV prevention, including male condoms and HIV tests, all biomedical innovations will require a behavioral component to support uptake, proper utilization, adherence, and maintenance of safe behaviors. Meanwhile, structural approaches and interventions should not replace behavioral and biomedical interventions, but rather should be incorporated into comprehensive responses to HIV prevention.

Focus group participants’ insights informed a successful adaption of *Connect ‘n Unite*, an intervention for Black MSM couples, to address the needs and concerns of Latino MSM and Latina transgender women couples. We modified session content to better address a range of issues salient to sexual and gender minority Latino/as, including the intersections homophobia and transphobia with anti-Latino prejudice, language barriers, immigration concerns and associated barriers to healthcare coverage, and the importance of culturally affirming care. Furthermore, we developed strategies for incorporating newly available biomedical approaches, such as PrEP and nPEP, into the adapted intervention. *Conectando Latinos en Pareja* (CLP), the adapted biobehavioral intervention, employs a social-ecological framework capturing biomedical approaches to better understand and address couple-based, institutional, cultural and social factors that fuel or mitigate the HIV epidemic among Latino MSM in same-sex relationships. Specific changes to the *Connect u Unite* intervention, as informed by the focus group results, include an established gender sensitivity training for facilitators and the referral agencies and/or the recommendation that they have openly LGBT staff serving the health needs of Latinos, as well as incorporation of biomedical strategies midway through the three-session intervention. In addition to expanding possibilities for sexual risk reduction, CLP could help identify a maximum number of HIV-infected persons through the different HIV testing mechanism built into the intervention, initiate ART to achieve long-term viral suppression and prevent new HIV infections through an integrated biomedical, behavioral, and culturally sensitive approach to HIV prevention and treatment. Overall, the intervention was met with great enthusiasm by both couples and providers.

In collaboration with our community stakeholders, we are currently applying for subsequent funding to assess the efficacy of such an integration approach before it is scaled up. This study provided substantial preliminary evidence to support the assumption that the adapted biobehavioral intervention could support the required medication adherence, retention to HIV testing, and improve sexual health promotion through risk reduction skills building among a vulnerable population. Any future success of the intervention will depend on ongoing community engagement and involvement, continued innovation to keep afloat with new prevention messages and tools, and the continued integration of social, cultural, and biomedical strategies.

### Limitations and Future Research

This study has some limitations. Our study aimed to assess how three biomedical interventions, nPEP, PrEP, and HIV self-testing kits, could be integrated into an existing couple-based HIV behavioral intervention. While this exploratory effort provided valuable insights, much remains to be done in terms of evaluating the efficacy of the adapted intervention. It will be important to evaluate this and other interventions that incorporate biomedical strategies to minimize risk compensation and maximize adherence. Given the potential for substance use to exacerbate sexual risk behaviors, future research should investigate the effect of alcohol and other substance use on PrEP and ARV adherence. Future studies should also explore strategies to maximize the cost-effectiveness of biomedical interventions. It should also be noted that our provider and community samples were relatively small, and the latter was comprised solely of couples; further research with larger samples, and that incorporate the insights of both coupled and uncoupled Latino MSM, would provide a valuable extension of this work. Finally, while we included the voices of transgender women who expressed interest in the study, the adapted intervention might have to be further refined for this particular group. We understand that Latina transgender women are impacted by unique syndemic conditions, including disproportionate histories of incarceration, high unemployment rates, legal issues including name change, and a culturally inclusive intervention should be developed for this group. In addition, given that transgender women did express interest in participating in research on Latino MSM, we encourage providers who serve Latino MSM to ensure that services are affirming and sensitive to the needs of Latina transgender women who have sex with men.

## Conclusions

There is a dearth of science-based, culturally congruent interventions to prevent HIV among Latino MSM, particularly those who are predominantly Spanish-speaking and those in same-sex relationships. Biomedical tools, including nPEP, PrEP, and HIV self-testing kits, have been shown effective in reducing HIV incidence and addressing some challenges with access to care, including access to HIV testing. Research has also documented the need to address cultural, social and structural barriers to reduce HIV incidence among Latino MSM, including homophobia, HIV-related stigma, and poverty. Our study builds on these findings and incorporates biomedical, cultural and social approaches into a behavioral HIV prevention intervention for Latino gay couples. Overall, couples and providers recommended having access to and information about three biomedical approaches—nPEP, PrEP, and HIV self-testing kits—and supported integrating these approaches into the existing behavioral intervention. This work could be extended to other behavioral interventions with new biomedical tools, provided that researchers and practitioners adapted those interventions and sought out interactive, effective feedback from the targeted community. These findings contribute to the growing body of literature on the importance of biomedical and behavioral approaches to HIV prevention among vulnerable groups.
